# *Akkermansia muciniphila* and *Lactobacillus plantarum* ameliorate systemic lupus erythematosus by possibly regulating immune response and remodeling gut microbiota

**DOI:** 10.1128/msphere.00070-23

**Published:** 2023-06-27

**Authors:** Mengchen Guo, Mei Lu, Kun Chen, Rui Xu, Yumin Xia, Xingyin Liu, Zhi Liu, Qisha Liu

**Affiliations:** 1 The Fourth Affiliated Hospital of Nanjing Medical University, Nanjing Medical University, Nanjing, China; 2 Department of Pathogen Biology-Microbiology Division, Nanjing Medical University, Nanjing, China; 3 Department of Dermatology, The Second Affiliated Hospital, School of Medicine, Xi'an Jiaotong University, Xi'an, China; 4 Zhongda Hospital, Southeast University, Nanjing, China; 5 Key Laboratory of Pathogen of Jiangsu Province and Key Laboratory of Human Functional Genomics of Jiangsu Province, Nanjing Medical University, Nanjing, China; 6 The Laboratory Center for Basic Medical Sciences of Nanjing Medical University, Nanjing, China; University of Michigan-Ann Arbor, Ann Arbor, Michigan, USA

**Keywords:** SLE, renal function, gut microbiota, probiotics, *Akkermansia muciniphila*, *Lactobacillus plantarum*

## Abstract

Systemic lupus erythematosus (SLE), characterized by persistent inflammation, is a complex autoimmune disorder that affects all organs, challenging clinical treatment. Dysbiosis of gut microbiota promotes autoimmune disorders that damage extraintestinal organs. Modulating the gut microbiome is proposed as a promising approach for fine-running parts of the immune system, relieving systematic inflammation in multiple diseases. This study demonstrated that the administration of *Akkermansia muciniphila* and *Lactobacillus plantarum* contributed to an anti-inflammatory environment by decreasing IL-6 and IL-17 and increasing IL-10 levels in the circulation. The treatment of *A. muciniphila* and *L. plantarum* restored the intestinal barrier integrity to a different extent. In addition, both strains reduced the deposit of IgG in the kidney and improved renal function significantly. Further studies revealed distinct remodeling roles of *A. muciniphila* and *L. plantarum* administration on the gut microbiome. This work demonstrated essential mechanisms of how *A. muciniphila* and *L. plantarum* remodel gut microbiota and regulate the immune responses in the SLE mice model.

**IMPORTANCE:**

Several pieces of research have demonstrated that certain probiotic strains contribute to regulating excessive inflammation and restoring tolerances in the SLE animal model. More animal trials combined with clinical studies are urgently needed to further elucidate the mechanisms for the effect of specific probiotic bacteria in preventing SLE symptoms and developing novel therapeutic targets. In this study, we explored the role of *A. muciniphila* and *L*. *plantarum* in ameliorating the SLE disease activity. Both *A. muciniphila* and *L. plantarum* treatment relieved the systemic inflammation and improved renal function in the SLE mouse model. We demonstrated that *A. muciniphila* and *L. plantarum* contributed to an anti-inflammatory environment by regulating cytokine levels in the circulation, restoring the intestinal barrier integrity, and remodeling the gut microbiome, however, to a different extent.

## INTRODUCTION

Systemic lupus erythematosus (SLE), characterized by severe and persistent inflammation that leads to tissue damage in multiple organs, is a prototypical autoimmune disease resulting from loss of self-tolerance and sustained autoantibody production (1).

A complex combination of genetic, environmental, hormonal, and other immunoregulatory factors contributes to the pathogenesis of SLE (2 - 4). Recently, the gut microbiota has been reported to trigger symptoms and progression of SLE. Changes in multiple bacteria taxonomies, including *Ruminococcus*, *Lactobacillus*, *Akkermansia*, and *Bacteroides fragilis*, were observed in SLE animal models or patients (5 - 8). Emerging evidence showed microbiota dynamics play a critical role in lupus pathogenesis in lupus-prone Murphy Roths Large (MLR)/Mp-Faslpr (lpr) mice. For example, several studies have reported an increase in *Lactobacillus* in SLE mice compared with the control mice (9, 10). Conversely, Zhang et al. reported a decrease in *Lactobacillaceae* in the MRL/lpr mouse model versus healthy controls (11). Meanwhile, *Akkermansia muciniphila* significantly decreased from the pre-disease stage to the diseased stage in mice (9).

Currently, probiotics have been experimentally and mechanically investigated for their possible eﬀectiveness in treating cancer, metabolic diseases, and autoimmunity diseases, including SLE (12). Mardani et al. showed that administering the probiotics *Lactobacillus delbrueckii* or *L. rhamnosus* to a pristane-induced SLE mouse model was able to prevent the initiation or the progression of the SLE disease (12). Luo et al. observed a striking effect of *Lactobacillus* spp. administration in ameliorating lupus nephritis in MRL/lpr mice (9). *L. plantarum* is a lactic acid bacterium with particular capabilities of producing diverse and potent bacteriocins, which have antibacterial properties (13). Moreover, Cabana-Puig et al. described *Lactobacillus* spp. act in synergy to attenuate splenomegaly and lymphadenopathy in lupus-prone MRL/lpr mice (14). To date, a body of evidence was constituted on the role of *L. plantarum* in medical cases such as diarrhea prevention, cholesterol lowering, and reduction in irritable bowel syndrome symptoms (15 - 17). In addition, *A. muciniphila* was considered one of the most promising candidates as probiotics have an essential value in improving the host metabolic functions and immune responses (18, 19). Hänninen et al. found that *A. muciniphila* would remodel gut microbiota and control islet autoimmunity in non-obese diabetic mice (20).

Our previous study has shown that the genera *Lactobacillus* and *Akkermansia* were enriched in the glucocorticoid-treated SLE patients (7). We have recently demonstrated that *L. plantarum* could restore intestinal permeability and regulate immunity-related pathways in Drosophila (21). In the present study, we tested a hypothesis that treating with *A. muciniphila* and *L. plantarum* might ameliorate the SLE disease activity by regulating the gut microenvironment and immune response in a classical SLE mouse model.

## MATERIALS AND METHODS

### Bacteria strains and growth condition

*A. muciniphila* (ATCC BAA-835) was purchased from Biobw (China). *L. plantarum* used in this study was obtained from our lab (21). *L. plantarum* and *A. muciniphila* were cultured on the de Man, Rogosa, and Sharpe (MRS) medium and Brain Heart Infusion medium with 2 g/L mucoprotein, respectively. Both strains were cultured at 37°C under anaerobic condition.

### Animal and experimental groups

Female MRL/lpr mice were originally obtained from Dr. Qian Zhang of NHC Key Laboratory of Antibody Technique (Nanjing Medical University). All animals were bred and maintained in a specific pathogen-free facility according to the requirements of the Institutional Animal Care and Use Committee at Nanjing Medical University (IACUC 1812014). The mice were cultured in a standard 12 h light/dark cycle, with controlled temperature (22 ± 2°C), and given water and food *ad libitum*.

**Fig 1:**
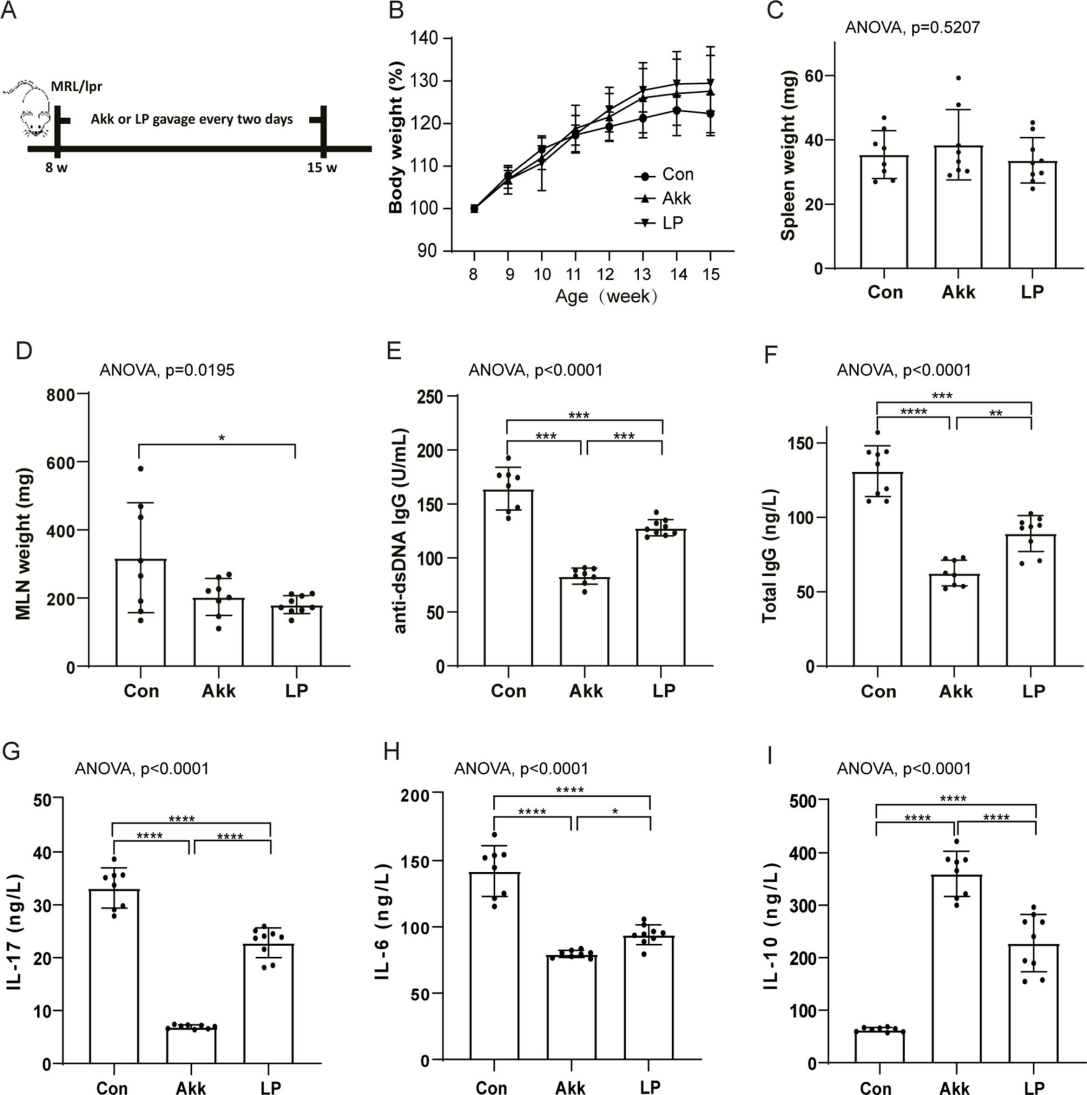
*A. muciniphila* and *L. plantarum* treatment ameliorated inflammation. (A) Study design: female SLE-related MRL/lpr mice (*n* ≥ 13 in each group) were fed with *A. muciniphila* (Akk), *L. plantarum* (LP), or control PBS (Con) every 2 days from 8 weeks to 15 weeks old. (B) Body weight variations from 8 weeks to 15 weeks old. (C–F) The measurement of spleen weight (C), mesenteric lymph node weight (D), anti-dsDNA IgG(E), and serum total IgG (F). (G–I) The measurement of serum cytokines, including IL-17 (G), IL-6 (H), and IL-10(I) at 15-week old. *P* values (*P < 0.05, **P < 0.01, ***P < 0.001, and ****P < 0.0001) are shown.

Forty-one female MLR/lpr mice were randomly divided into the following three groups: SLE controls (Con; *n*＝13), SLE mice treated with *L. plantarum* group (LP; *n*＝14), and SLE mice treated with *A. muciniphila* group (Akk; *n*＝14). *A. muciniphila* and *L. plantarum* were suspended using a sterile phosphate-buffered saline (PBS) solution and diluted to obtain a concentration of 1 × 10^9^ CFUs. The SLE controls were treated with sterile PBS. Probiotics gavage was performed every 2 days from the 8th week to the 15th week (Fig. 1A). Mice were euthanized at 15-week old, and spleen weight was measured. Bodyweight was measured each week after treatment began.

### Quantification of serum cytokines

Blood was collected by cardiac puncture after the euthanasia of mice. Serum was stored at −80°C for later analysis. Cytokine levels, including interleukin (IL)-17, IL-6, and IL-10, were analyzed using enzyme-linked immunosorbent assay (ELISA) sensitivity mouse multiplex assay (Jiyinmei Bio, China), according to the manufacturer’s instructions. In addition, serum anti-double-stranded DNA (dsDNA) IgG and total IgG were measured using standard ELISA protocols (Cat#JYM1061Mo, Cat#JYM0031Mo, Jiyinmei Bio, China).

### Assessment of colitis severity and histological scoring

The distal colon tissue was fixed with 4% paraformaldehyde for 24 h, then embedded in paraffin and cut into sections 5 µm thickness for hematoxylin-eosin (HE) staining at Servicebio company (China). The HE staining images were scanned using Pannoramic MIDI Digital Section Scanner (3DHISTECH, Hungary), and the images were analyzed by Case View software. The histological scores of the colon were assessed given a score range from 0 to 4 as previously described parameters: inflammation, depth of inflammation, crypt damage, loss of goblet cells, and thickness of the colon wall (22, 23).

### Renal function

Urine samples were tested two times for proteinuria. Mice with 8-week old and 15-week old were placed in individual metabolic cages for urine collection for a period of 12 h, respectively. All samples were stored at −20°C until being processed simultaneously. Urine samples were analyzed using ELISA kit to measure total protein level (Cat#RJ17462, Renjie Bio, China). Meanwhile, serum creatinine and blood urea nitrogen (BUN) were also measured using ELISA kit (Cat#RJ17464, Cat#RJ17469, Renjie Bio, China).

The HE staining of kidneys was also conducted at Servicebio company (China). One kidney section per mouse was evaluated. Each glomerulus was examined at 400× magnification and scored from 0 (normal) to 4 (severe) based on the glomerular size and lobulation, presence of karyorrhectic nuclear debris, capillary basement membrane thickening, and the degree of mesangial matrix expansion and mesangial cell proliferation as described (24). All measurements and analyses were scored in a blinded fashion by two pathologists. Images were acquired using BX53 Light Microscope (Olympus, Japan).

### Immunofluorescence analysis

Kidney sections were stained for IgM, IgG, and IgA with goat polyclonal antibody anti-rabbit IgM (1:500 dilution; Cat#ab190369, abcam, UK), anti-rabbit IgG (1:500 dilution; Cat#ab150113, abcam, UK), or anti-mouse IgA (1:500 dilution; Cat#sc373823, Santa Cruz Biotechnology, USA) antibody as previously described (25). In addition, to assess the tight junctional permeability of the colon, the expression of claudin-7 immunoreaction was performed using the standard protocols (26). Images were acquired using an LSM 800 laser scanning confocal microscope (Zeiss, Germany). Fluorescence intensity was scanned and quantified by ImageJ software.

### Fecal sample collection and DNA extraction

Fecal samples were collected in a sterile stool container, frozen at −80°C within 2 h of sample collection. About 100 mg stool samples were used to extract total genome DNA following the protocol of the DNA extraction kit (Cat#DP328, Tiangen, China). The concentration and purity of the extracted bacterial DNA were detected using Qubit 2.0 Fluorometer (Thermo Scientific, USA). DNA quality and quantity were determined by agarose gel electrophoresis.

### 16S rRNA gene amplicon sequencing and analysis

Polymerase chain reaction (PCR) was performed to produce V4 regions of the 16S rRNA gene using the conserved primers 515F (5′-GTGCCAGCMGCCGCGGTAA-3′) and 806R (5′-GGACTACHVGGGTWTCTAAT-3′), and no template DNA reaction was used as a negative control. PCR products were purified using the GeneJET Gel Extraction Kit (Thermo Scientific, USA). Following manufacturer’s recommendation, sequencing libraries were generated using the Illumina TruSeq DNA PCR-Free Library Preparation Kit (Illumina, USA). PCR fragments were sequenced in the Illumina NovaSeq platform (Novogene, China).

Bioinformatics analysis of 16S rRNA gene amplicons was performed by Qiime2 (version 2020.8.0) (27). Briefly, fastq reads were processed by the dada2 program, and dada2 denoise-paired commands were used to delete the low-quality ones. Dada2 generates unique features that could be compared between different studies. The taxonomy of these features was assigned to the silva reference database (version 138) classifier with 99% similarity (28). At each taxonomy level, the taxons with relative abundance less than 0.0001 were filtered out. Determination of alpha and beta diversities was conducted by R packages vegan.

### Functional analysis

The functional capacity of the gut microbial community was predicted using PICRUSt2. Predicted functional genes were categorized into MetaCyc pathways. The relative pathway abundance change (assigned as deta) between the pre- and post-treatment for each mouse was calculated. The deta value for each treatment was compared with the Wilcoxon rank-sum test. MetaCyc pathway changes with fdr adjusted *P* values <0.05 were determined as significant.

### Microbial network analysis

The co-occurrence microbial network of each experiment was conducted by Spearman correlation based on the relative abundance of each genus. The correlation with *P* value < 0.004 and correlation value > 0.8 was represented in the figure.

### Statistical analysis

The student *t*-test was used for the comparison of two groups. For comparison of more than two groups, a single-factor analysis of variance was performed. Kruskal-Wallis test was applied for data that did not meet the normal distribution. All the measured data were displayed as means ± SD, and the analysis was performed using GraphPad Prism software. Significance was defined as: * *P* < 0.05; ** *P* < 0.01; and *** *P* < 0.001. The bacterial taxonomic analysis between any two groups was conducted using the two-sided Wilcoxon rank-sum test performed by the R program.

## RESULTS

### *A. muciniphila* and *L. plantarum* treatment relieved systemic inflammation

To determine the effects of probiotics on active disease in MRL/lpr mice, female mice were gavaged with *A. muciniphila* or *L. plantarum* starting from 8 weeks of age to 15 weeks old (Fig. 1A). The probiotics treatment increased body weight as expected (Fig. 1B), whereas the spleen weight did not change (Fig. 1C). It is worth noting that mesenteric lymph node weights were significantly decreased with LP treatment compared with SLE controls (Fig. 1D). The production of antinuclear antibodies (ANA) is the immunological hallmark of SLE (29). The anti-dsDNA is one of a group of ANA. We next assessed the serum anti-dsDNA titers. The results showed that both Akk and LP-treated groups had significantly lower levels of serum anti-dsDNA than SLE controls, especially the Akk-treated group (Fig. 1E). In addition, mice treated with both probiotics secreted lower levels of serum IgG, while the decreased effect of the Akk-treated group was much more apparent (Fig. 1F).

It has been widely reported that overexpression of cytokines plays a critical role in SLE pathogenesis (30). The pro-inflammatory cytokines IL-17 secreted by autoimmune Th17 cells have been shown to facilitate SLE development (31). Consistent with the importance of IL-17 in SLE, polarizing Th17 cells by stimulation with IL-6 can acquire pathogenicity and elicit SLE (32). As might be expected, the serum expression of IL-17 and IL-6 was significantly lower in probiotics-treated groups, especially in the Akk-treated group (Fig. 1G and H). IL-10 has been shown to protect against SLE by suppressing pathogenic Th1 responses, including IFN-γ-mediated autoantibody production and renal inflammation (33). Of particular interest, the anti-inflammatory cytokine IL-10 was increased in probiotics-treated mice compared to SLE controls, particularly in the Akk-treated group (Fig. 1I). These results indicated that *A. muciniphila* and *L. plantarum* administration indeed relieved inflammatory response in the SLE model.

### *A. muciniphila* and *L. plantarum* treatment improved renal function

**Fig 2:**
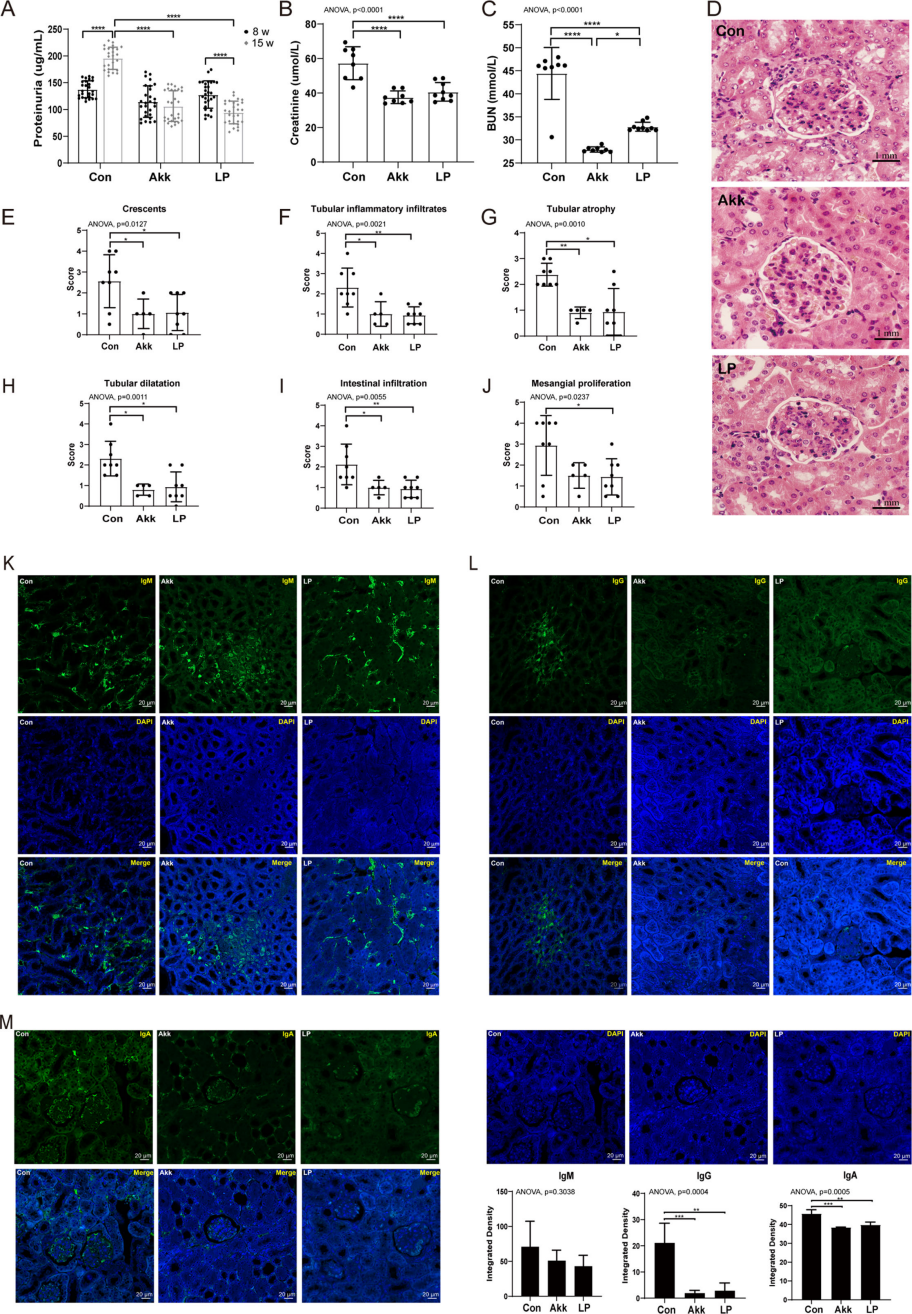
*A. muciniphila* and *L. plantarum* treatment improved renal function. (A–C) Analysis of renal function by measuring proteinuria (A), serum creatinine (B), and blood urea nitrogen (C), *n* ≥ 13. (D–J) Representative HE-staining images (D) and histological scores (E–J) of the renal tissues at 15-week old, *n* ≥ 5. (K–M) Immunofluorescent staining of IgM (K), IgG (L), and IgA (M) counterstained with DAPI in the renal tissues, *n* ≥ 4. The below panel shows the arithmetic mean intensity ratio of probiotic to controls. *P* values (*P < 0.05, **P < 0.01, ***P < 0.001, and ****P < 0.0001) are shown.

The disease phenotype in MRL/lpr mice resembles human SLE, which is characterized by an increased level of proteinuria and progressive immune complex glomerulonephritis (34). Lupus nephritis is the most common cause of renal injury in SLE and the most important predictor of mortality in patients with SLE (35). Next, we determined the renal function by measuring the proteinuria and the kidney histopathology scores. Compared with the SLE controls, mice in probiotics-treated groups exhibited improved renal physiology characterized by decreased levels of proteinuria (Fig. 2A), creatinine (Cr) (Fig. 2B), and BUN (Fig. 2C). Moreover, the administration of both Akk and LP significantly ameliorated the kidney injury characterized by the reduced scores in crescents, tubular inflammatory infiltrates, tubular atrophy, tubular dilatation, and intestinal infiltration according to the renal histopathology scores (Fig. 2D through I). Besides, the mesangial proliferation score significantly decreased in LP-treated group but decreased in Akk-treated group with a marginal significance (*P* = 0.059) (Fig. 2J). IgG-autoantibodies are major immune deposits in the kidney and trigger lupus nephritis (36). Both Akk and LP relieved the IgG-autoantibodies rather than the IgM-autoantibodies deposited in the kidney (Fig. 2K and L). Moreover, the expression of IgA protein was also dramatically decreased after probiotics treatment (Fig. 2M). Thus, *A. muciniphila* and *L. plantarum* administration could improve the renal function in the SLE model.

### *A. muciniphila* and *L. plantarum* treatment exerted protective effects in the intestinal barrier integrity

**Fig 3:**
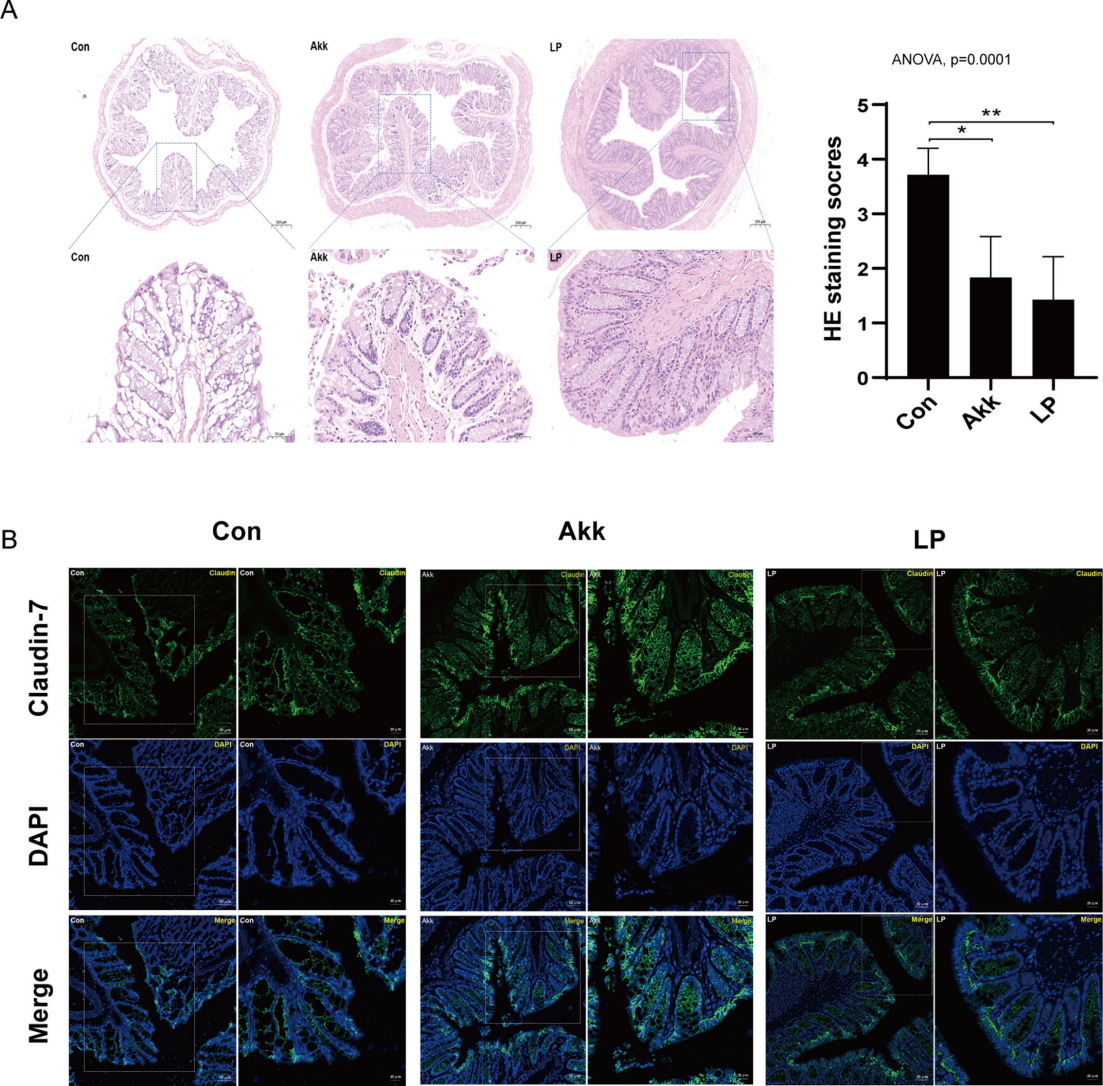
*A. muciniphila* and *L. plantarum* treatment ameliorated intestinal inflammation and barrier integrity. (A) Representative HE-staining images (left) and histological scores (right) of the colonic tissues, *n* ≥ 6. (B) The tight junctional permeability of the colon was evaluated by claudin-7 expression using immunofluorescence. *P* values (*P < 0.05 and **P < 0.01) are shown.

The gastrointestinal symptoms were reported to occur in >50% of SLE patients, and lupus enteritis was possibly identified as an initial manifestation in SLE (37, 38). The histological examination showed that both probiotics restored the colonic histomorphology to a certain extent (Fig. 3A). Impressively, the epithelial damage was enhanced in control mice, which presented massive loss of goblet cells and crypt. Histopathological scores confirmed that significantly higher scores in control group versus probiotics-treated group according to the previously described criteria (22, 23). The high score of control group was due to the crypt being nearly destroyed. All the colonic HE staining was listed in the supplementary information . To assess the intestinal permeability, we examined the effects of probiotics upon tight junction structure stained with the marker claudin-7 (Fig. 3B). The immunofluorescence analysis demonstrated that claudin-7 redistributes in the cytoplasm due to the dysplasia of intestinal crypt in SLE control group. Nevertheless, there were significantly increased changes in the tight junction structure of colonic epithelial cells after the probiotics intake. The claudin-7 was localized to the integrity intestinal epithelium in the Akk and LP group, which extended from the base to the tip of the colonic crypts. Taken together, our data showed that the *A. muciniphila* and *L. plantarum* help maintain intestinal function and barrier integrity in the SLE model.

### *A. muciniphila* and *L. plantarum* treatment altered the structure and diversity of the gut microbiota

**Fig 4:**
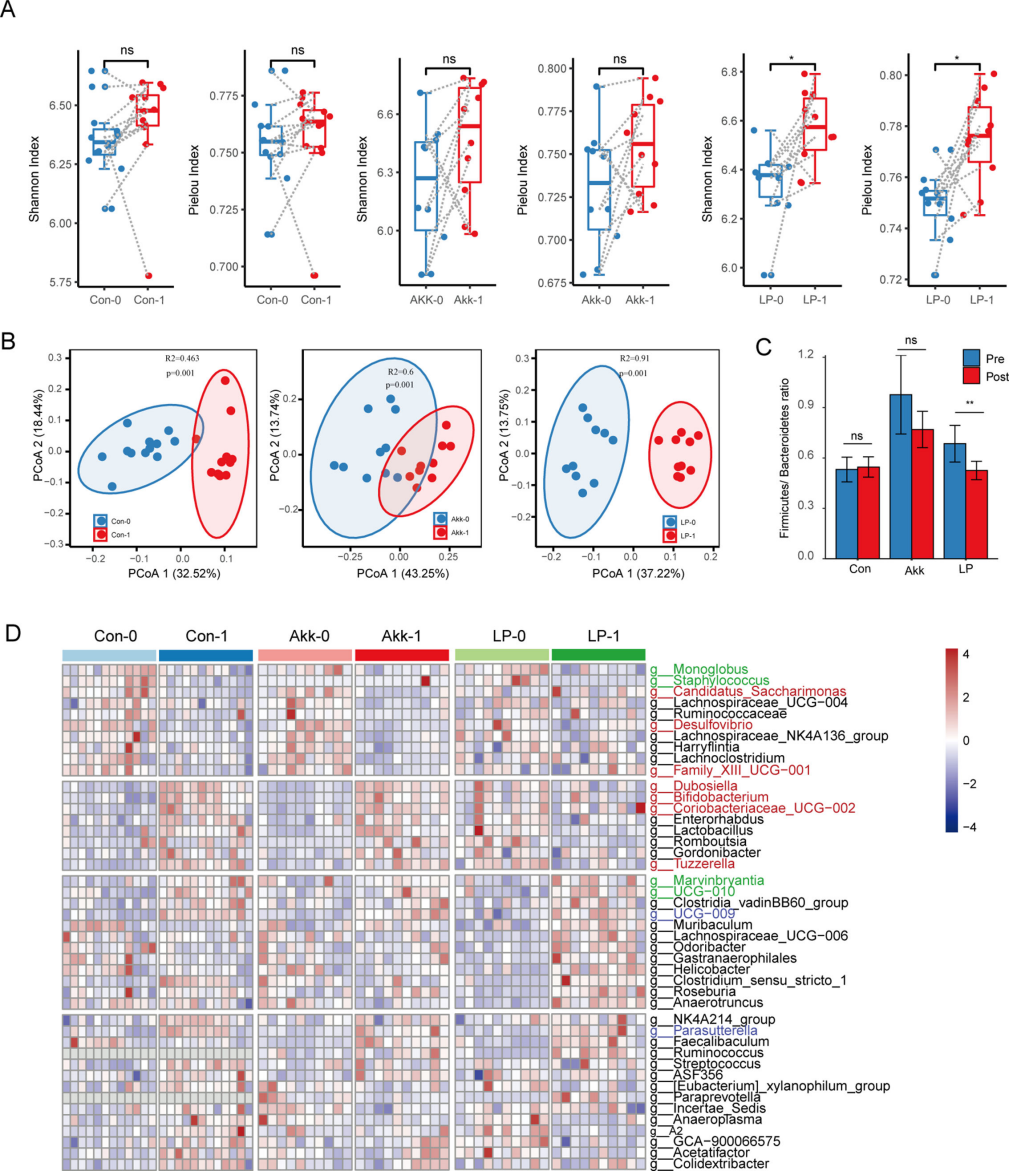
*A. muciniphila* and *L. plantarum* treatment changed the structure of gut microbiota. (A) The alpha diversity of the pre- and post-treatment samples in each treatment group. (B) The bacterial beta diversity based on the Bray-Curtis distance in the gut microbiota from all experimental groups. (C) The ratio of *Firmicutes*/*Bacteroidetes* in each group. (D) The genera with differential abundance in control, LP-treated, or Akk-treated groups. The codes 0 and 1 represent pre-treatment and post-treatment, respectively. Con, control group. Green, red, and blue labels indicate common differential genera in the control and LP-treated group, control, and Akk-treated group, and common differential genera in all three groups, respectively.

Increasingly studies demonstrated that dysbiosis of the gut microbiome may be involved in SLE development and progression (39). We analyzed fecal DNA isolated from all experimental mice groups to determine the dynamics of gut microbiota before and after probiotic treatment. To explore the bacterial composition alteration of probiotic treatment, we evaluated multiple ecological parameters, including Shannon and Simpson diversity (the combined parameters of richness and evenness), Pielou evenness (to show how evenly the individuals in the community are distributed over different operation taxonomic units [OTUs]), Chao richness (an estimate of a total number of OTU present in the given community), and Richness (the number of OTU). In the LP-treated group, the Shannon and Pielou index was significantly increased after the treatment compared with the pre-treatment samples (Fig. 4A), indicating an increased evenness of the gut microbial community after the treatment of LP. In contrast, the Akk treatment demonstrated no influence on alpha diversity. To be noticed, the number of taxonomies represented by the index of Richness and Chao was increased for the post-treatment samples in the control group (Fig. S2A), which might represent the microbiota dynamic during the disease progression (7). The administration of both *A. muciniphila* and *L. plantarum* reversed this tendency (Fig. S2A).

Analysis of Bray-Curtis distance based on the OTU-level composition revealed different microbiome structures between the pre-treatment and post-treatment samples in each experimental group (Fig. 4B). Moreover, the administration of LP was able to decrease the *Firmicutes*/*Bacteroidetes* ratio (Fig. 4C) as a result of a decrease in the proportion of *Firmicutes* and an increase in the proportion of *Bacteroidetes* (Fig. S2B).

Furthermore, differential bacterial genera between the pre-treatment and post-treatment samples were defined with paired Wilcoxon rank-sum test (Fig. S2C). There were 21 genera with different proportions in the control group, representing the microbes that altered during the progress of the disease. Of these genera, six and nine genera were also changed upon the administration of Akk and LP, respectively (Fig. 3D). *Parasutterella*, for example, was increased consistently in all three groups. *Parasutterella* was a harmful bacterium that increased with age in mice (40). The abundance of a short-chain fatty acid producer, *Faecalibaculum*, was reversed by the Akk and LP administration. In addition, LP treatment specifically increased the accumulation of butyrate-producing *Lachnospiraceae*, i.e., *Lachnospiraceae*_UCG-006 and *Roseburia*, which is beneficial for the intestinal barrier. Multiple species of *Roseburia* were enriched in healthy samples compared with SLE patients (6). These results indicated the role of *L. plantarum* in improving the gut barrier integrity.

### *A. muciniphila* and *L. plantarum* treatment exerted different impacts on gut microbiota

**Fig 5:**
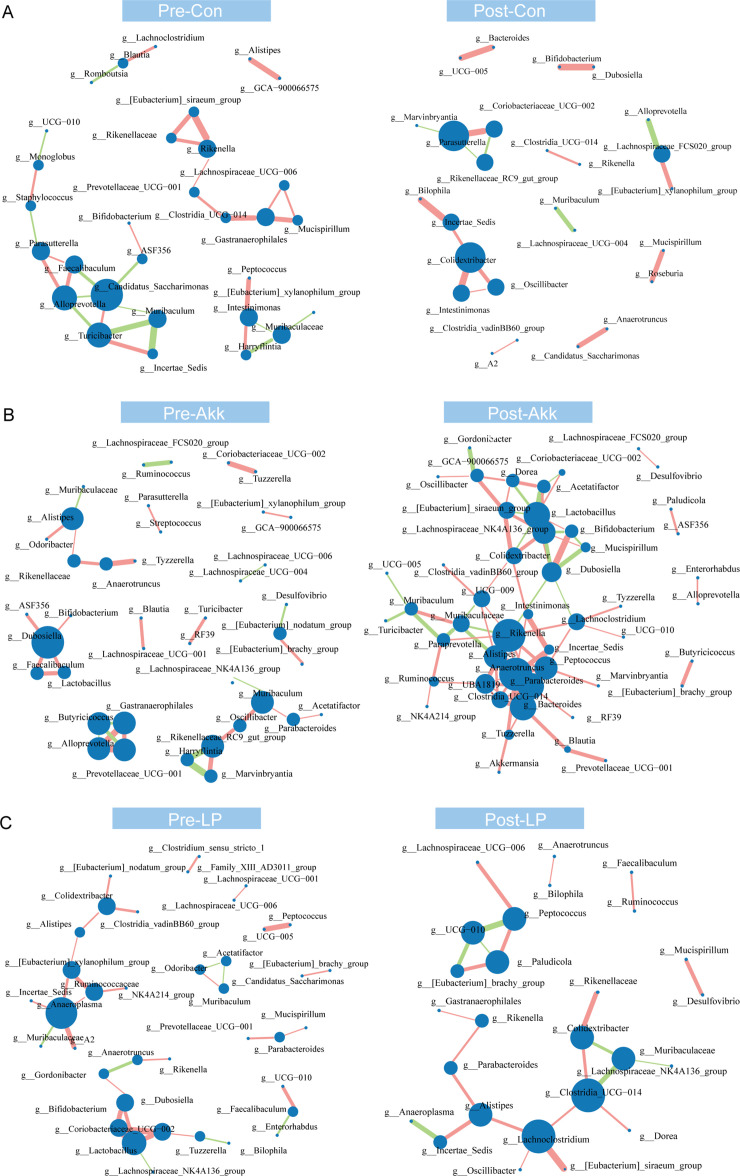
Co-occurrence network of microbiota in the control (A), Akk-treated (B), and LP-treated (C) groups. The circle size represents the degree of nodes, and the density of lines between circles represents the Spearman coefficient. The green and red links stand for negative or positive interactions, respectively. Con, control group.

To further explore the effects of Akk and LP administration on the gut microbiota, we assessed the interactions among the genera in each experimental group. In the control and LP-treated group, the complexity of network structure decreased slightly in the post-treatment mice (Fig. 5). However, in the Akk-treated group, the complexity increased dramatically with a sharply increased number of edges and neighborhood connectivity (Fig. 5; Fig. S3A). These results indicated a notable impact of *A. muciniphila* treatment on the microbial community.

**Fig 6:**
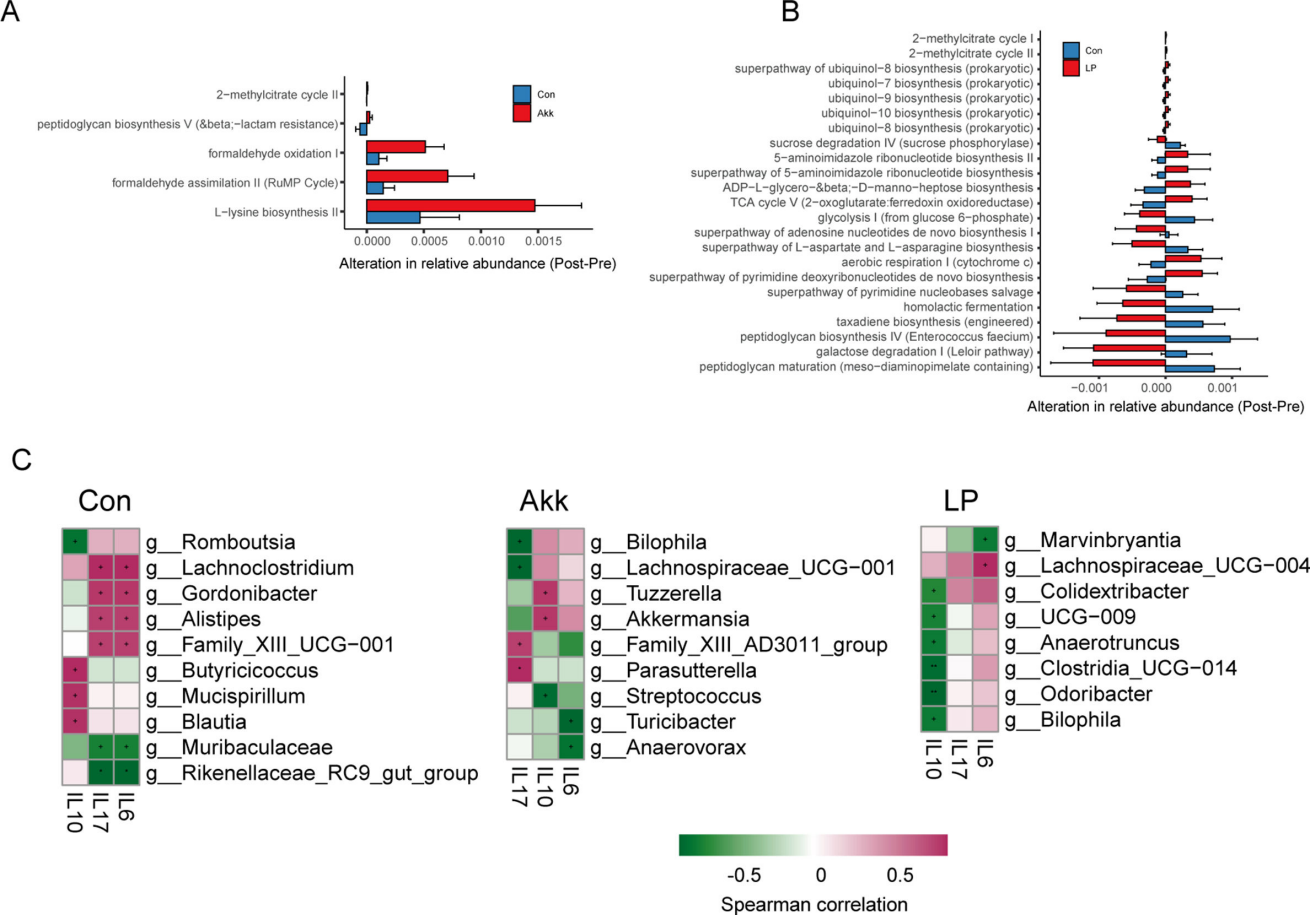
Functional alteration of microbes and their correlation with cytokines. (A) The differential alteration of MetaCyc pathways after being treated with Akk compared with controls. (B) The differential alteration of MetaCyc pathways after being treated with LP compared with controls. (C) The Spearman correlation between genera and cytokines in each of the experimental groups. Correlation coefficients and fdr (+ fdr < 0.05, * fdr < 0.01, and ** fdr < 0.005) were shown. Con, control group. The red and green cells of the heatmap indicate positive and negative correlations, respectively.

Moreover, to predict whether *A. muciniphila* and *L. plantarum* could modulate the microbial metabolic function of SLE mice, we conducted a functional metagenomics prediction based on 16S rRNA sequencing using the PICRUSt2 (41). We calculated the alteration of relative abundance of each metabolic pathway between the pre- and post-treatment samples. Compared with the control group, the Akk treatment altered a small number of metabolic functions. Most of them were changed in the same direction as the control group (Fig. 6A). Impressively, the LP treatment differentially changed multiple metabolic pathways in the opposite direction compared with the control group (Fig. 6B). For example, the tricarboxylic acid cycle (TCA) cycle was decreased in the post-PBS-treated samples but increased in the post-LP-treated mice. The TCA cycle was an essential pathway for biosynthesis and energy metabolism. Abnormal *T* cell activation and apoptosis are involved in the pathogenesis of SLE, which is highly energy dependent (42, 43). Similarly, the aerobic respiration pathway, one of the most critical energy production processes, increased after the LP treatment.

Furthermore, to find the potential bacteria associated with the immune disorder in SLE, we conducted a correlation analysis between microbial genera and cytokine levels in each treatment group. As shown in Fig. 6C, *A. muciniphila* was positively correlated with the level of IL-10 in the Akk-treated group. Conversely, no correlation was observed between LP and any cytokines in the LP-treated group. These results were consistent with that reported by Guo et al. in SLE patients that *A. muciniphila* but not *L. plantarum* was extensively associated with cytokines (7).

In summary, as per the above analysis, *A. muciniphila* and *L. plantarum* treatment may reduce the SLE symptoms in distinct pathways to mediate the interaction of host and microbiota.

## DISCUSSION

**Fig 7:**
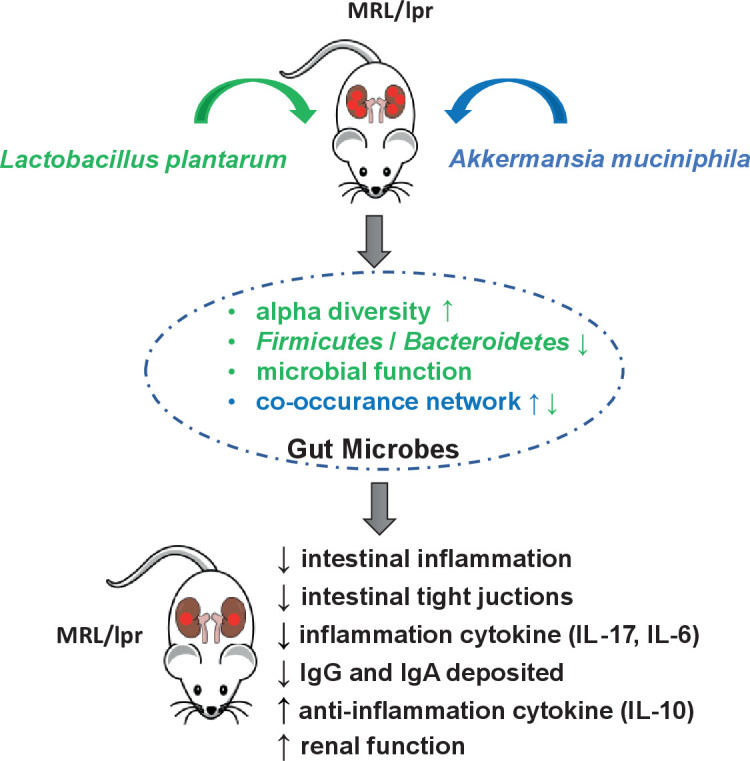
A proposed working model of *A. muciniphila* and *L. plantarum* administration both remodeled gut microbiota and regulated the immune responses in the MRL/lpr SLE mice model.

In the present study, we demonstrated for the first time that treatment with *A. muciniphila* and *L. plantarum* can improve the inflammation, intestinal tract, and renal damage occurring in an experimental SLE mice model. Both Akk and LP showed a protective role in the MRL/lpr mice, represented by reduced overall inflammation, intestinal tight junction, and improved renal function (Fig. 7). Herein, we suggest the critical role of gut microbiota manipulation in relieving the systemic symptoms in a mouse model of SLE.

Both *L. plantarum* and *A. muciniphila* strains are promising probiotics that benefit multiple diseases. *L. plantarum* is well known for its antibacterial property (44 - 46). In animal models and clinic samples, multiple strains of *L. plantarum* have been demonstrated to improve intestinal barrier integrity and modulate immune response (16, 47 - 49). *A. muciniphila* is an abundant member of the human intestinal microbiota (50) that can degrade mucin (51). *A. muciniphila* was reported to participate in the host immune regulation (52). Additional pieces of evidence have shown that *A. muciniphila* also enhances the integrity of the intestinal epithelial cells and the thickness of the mucus layer, thereby promoting intestinal health (53). In this study, we compared the protective roles and possible mechanisms of *A. muciniphila* and *L. plantarum* on MRL/lpr mice.

Firstly, we observed that *A. muciniphila* and *L. plantarum* had similar effectiveness in ameliorating the progression of SLE according to the inflammation cytokines and intestinal inflammation (Fig. 1 and 2). The administration of probiotics both promoted an anti-inflammatory environment, A ressing the expression of proinflammatory cytokines IL-6 and IL-17 while increasing the levels of anti-inflammatory cytokine IL-10 in circulation, especially *A. muciniphila* introduced more pronounced alterations (Fig. 1G through I). These results indicated a more vital function of *A. muciniphila* in immunity regulation than *L. plantarum*. In MRL/lpr mice, the increased intestinal permeability has been described (54). Our data showed that the intestinal epithelium was compromised in lupus mice. Treatment with *A. muciniphila* and *L. plantarum* restored mucosal barrier integrity (Fig. 3). However, lower cumulative scores characterized by crypt hyperplasia, epithelial injury, and inﬂammation were observed in *L. plantarum* treated mice compared with control and the *A. muciniphila* treated mice, indicating a better ability of *L. plantarum* to improve intestinal barrier integrity than *A. muciniphila*.

Dysbiosis of the gut microbiota is reported repeatedly to contribute to SLE in humans (6 - 9 - 55) and a lupus-like autoimmune disease in mice (9, 10, 54, 56). From the perspective of gut microbiome community restores, a couple of probiotics, including *A. muciniphila* and *L. plantarum* can recover gut microbiota imbalance (20, 57, 58). We found pretty diverse ways of microbiota remodeling between *A. muciniphila* and *L. plantarum*. *L. plantarum* administration significantly increased the alpha diversity of gut microbiota (Fig. 4A), which was reduced in SLE mice and patients (6, 10). Whereas SLE mice treated with *A. muciniphila* had no changes in the alpha diversity (Fig. 4A). In addition, the LP treatment altered the microbial metabolic function considerably compared with the PBS-treated group (Fig. 6B). However, Akk treatment only slightly remodeled the microbial metabolic function (Fig. 6A). On the other hand, Akk administration significantly increased the network complexity of the microbial community (Fig. 5B). Akk was found to be correlated with the level of cytokines (Fig. 6C), while the LP treatment did not. Hence, we deduced that *L. plantarum* may exert antibacterial function and have a better ability to improve intestinal barrier integrity. However, Akk might play a prominent role in immune regulation. This hypothesis was consistent with the changes in inflammatory factors (Fig. 1G through I) and previous studies. Multiple studies have reported the role of Akk in affecting Treg or Th17 cells. For example, Liu et al. have reported that Akk supplementation suppresses colon inflammation and increases the frequency of colonic RORγt+ Treg cells (59). Moreover, using a new approach to study epitopes and identify *T* cell receptors expressed by CD4+ Foxp3+ (Treg) cells specific for commensal-derived antigens. Kuczmus et al. found that antigens from Akk reprogram naïve CD4+ T cells to the Treg lineage and expand pre-existing microbe-specific Tregs (60). Additionally, a recent study revealed that Akk promotes the accumulation of Th1 and Th17 cells in the gut (61).

In this study, we performed probiotic treatment at 8 weeks old, when the MRL/lpr mice started to develop lupus-like symptoms. However, in clinical cases, patients with developed SLE should be considered. Studies of SLE patients showed intense alteration in the gut microbiome (6, 7). Moreover, a recent clinical trial performed in lupus patients with developed SLE and gastrointestinal symptoms revealed that supplementing synbiotics could improve gut microbiota and systemic inflammation (62). Future studies to conduct probiotic treatment in the MRL/lpr mice with progressed SLE will provide additional valuable evidence for clinical application.

In brief, the present study demonstrated an essential role of *A. muciniphila* and *L. plantarum* in improving the inflammation and renal damage of the MRL/lpr mice. Future studies are necessary to further explore the molecular mechanisms involved.

## Data Availability

The raw metagenomic sequencing data generated in this study have been deposited in the CNGB Sequence Archive (CNSA) of China National GeneBank DataBase (CNGBdb) with accession number CNP0002181.

## SUPPLEMENTAL MATERIAL

Fig. S1 msphere.00070-23-s0001.pdf Representative all the colonic HE-staining images.

Fig. S2 msphere.00070-23-s0002.pdf The gut microbiota changes in the control, *A. muciniphila* and *L. plantarum* treated group. (A) The alpha diversity of Richness, Chao1, and Simpson of the pre- and post-treatment samples in each treatment group. (B) The relative abundance of bacterial phylum in all experiment groups. (C) Veen plot of differential bacterial genera in control, pre-treatment and post-treatment samples.

Fig. S3 msphere.00070-23-s0003.pdf The network statistics for the microbial community network of the pre- and post-treatment group of control, Akk-treated, and LP-treated mice.

ASM does not own the copyrights to Supplemental Material that may be linked to, or accessed through, an article. The authors have granted ASM a non-exclusive, world-wide license to publish the Supplemental Material files. Please contact the corresponding author directly for reuse.
