# Supplementary figures and images for: Akkermansia muciniphila and Lactobacillus plantarum ameliorate systemic lupus erythematosus by possibly regulating immune response and remodeling gut microbiota
Source: mSphere. 2023 Jun 27;8(4):e00070-23. doi: 10.1128/msphere.00070-23 (PMC10449527; doi:10.1128/msphere.00070-23)

**Con**

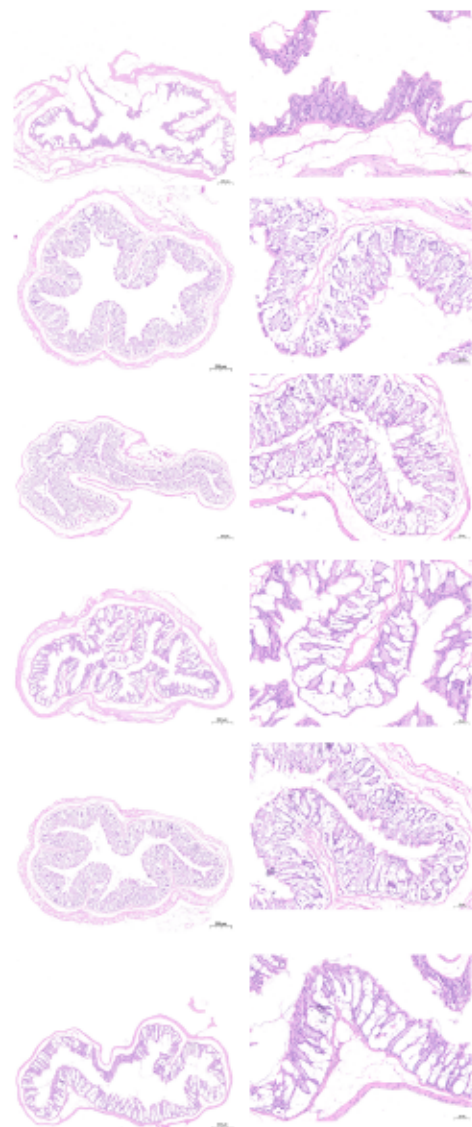

**Akk**

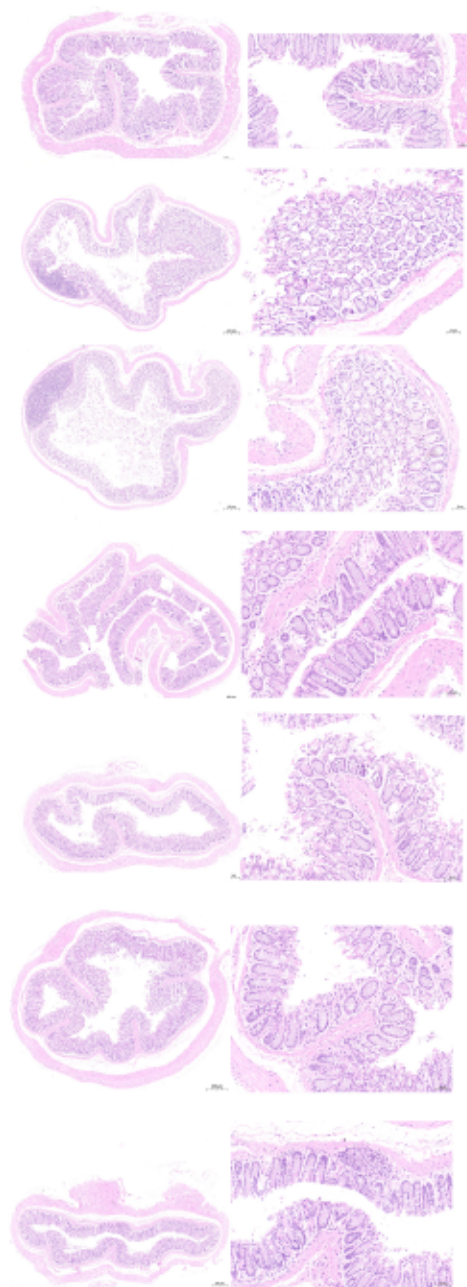

**LP**

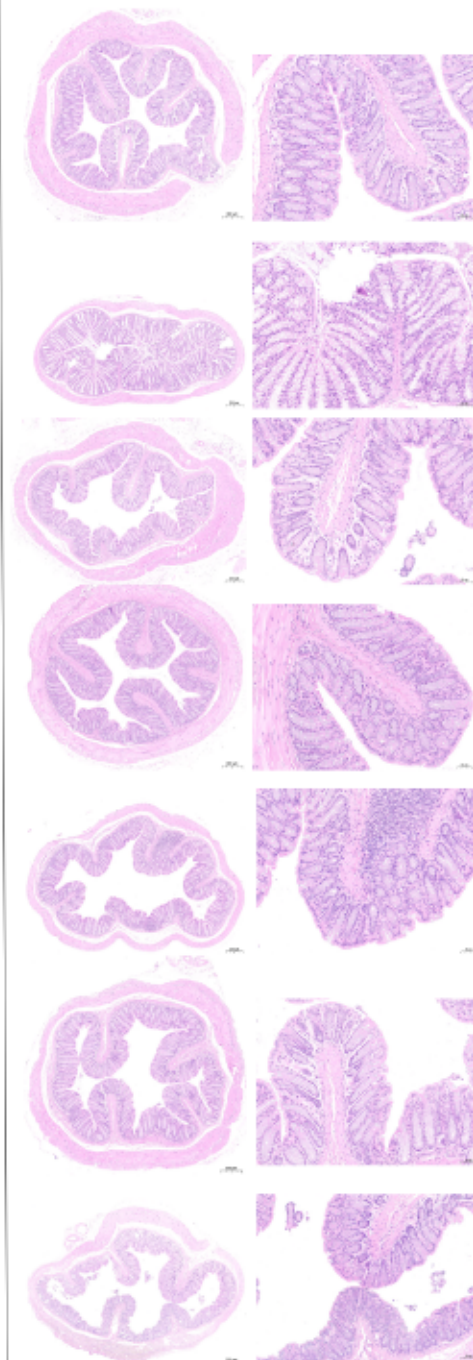

Supplement: Fig. S1 — Representative all the colonic HE-staining images. [file msphere.00070-23-s0001.pdf]

A

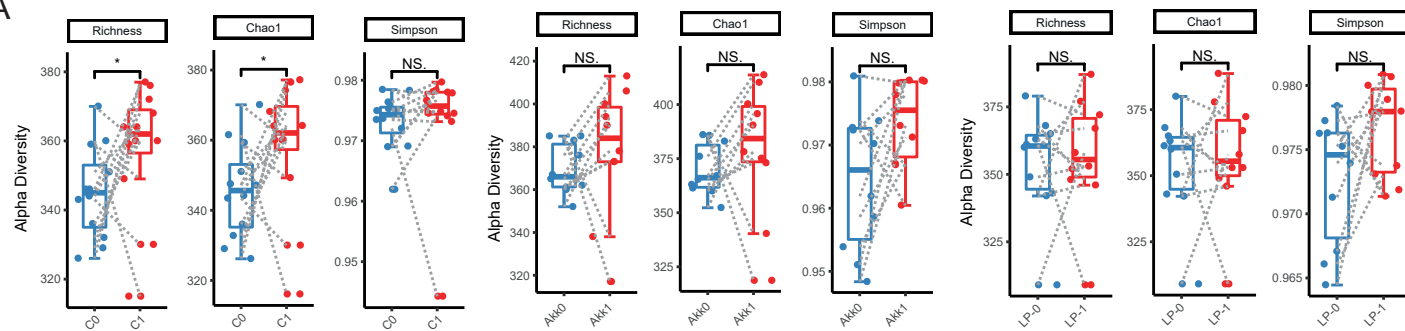

B

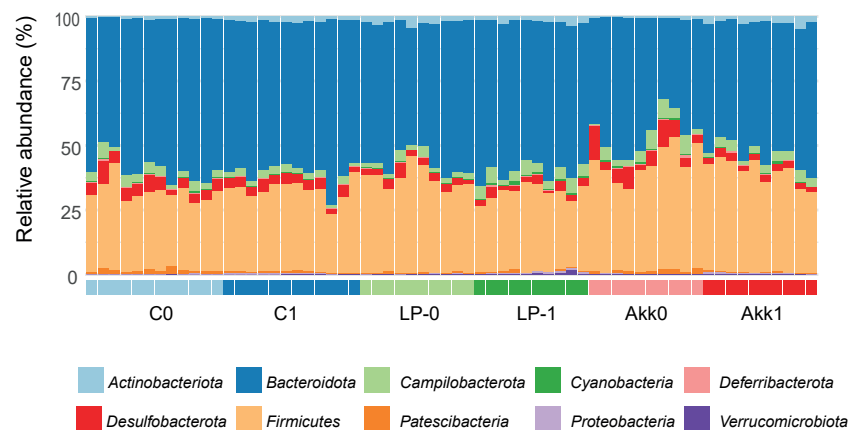

C

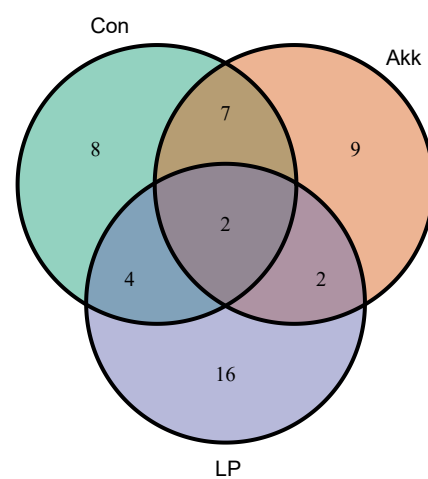

Supplement: Fig. S2 — The gut microbiota changes in the control, A. muciniphila and L. plantarum treated group. (A) The alpha diversity of Richness, Chao1, and Simpson of the pre- and post-treatment samples in each treatment group. (B) The relative abundance of bacterial phylum in all experiment groups. (C) Veen plot of differential bacterial genera in control, pre-treatment and post-treatment samples. [file msphere.00070-23-s0002.pdf]

A

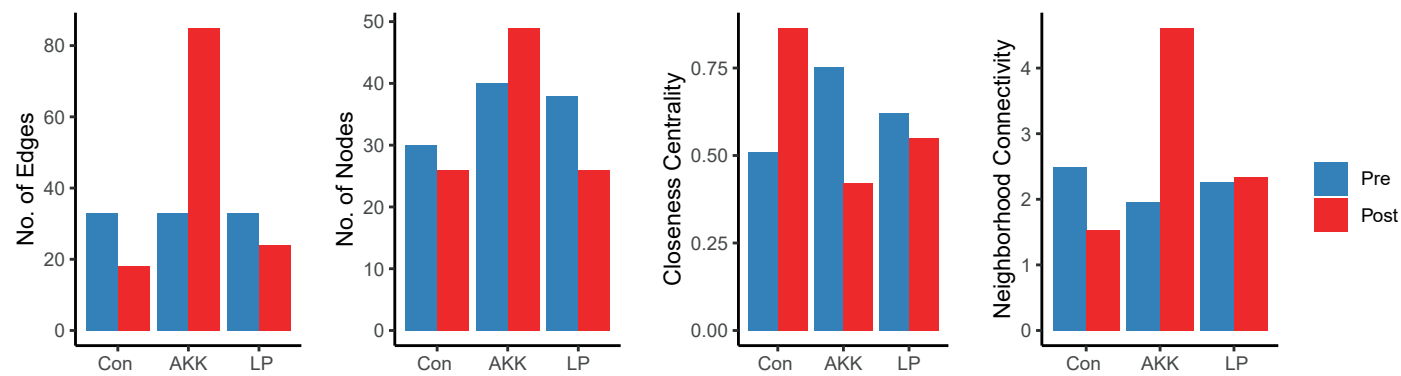

Supplement: Fig. S3 — The network statistics for the microbial community network of the pre- and post-treatment group of control, Akk-treated, and LP-treated mice. [file msphere.00070-23-s0003.pdf]
